# Artificial intelligence-based prediction of procedural success in transcatheter mitral valve edge-to-edge repair

**DOI:** 10.1186/s44156-026-00134-2

**Published:** 2026-09-07

**Authors:** Mathias Claeys, Neil Fam, Xiaojiao Xiao, Tejas Vyas, Mohsena Chowdhury, Gordon Moe, Sami Alnasser, Patrick Prud’homme, Anas Merdad, Akshay Bagai, Kim A. Connelly, Jeremy Edwards, Guanghui Wang, Géraldine Ong

**Affiliations:** 1 Division of Cardiology, St. Michael’s Hospital, University of Toronto, Toronto, ON Canada; 2 Toronto Metropolitan University, Toronto, ON Canada; 3 Division of Cardiology, Department of Medicine, St. Michael’s Hospital, University of Toronto, Toronto, ON Canada

**Keywords:** Artificial intelligence, Deep learning, Mitral valve edge-to-edge repair, Outcome prediction

## Abstract

**Background:**

Patient selection for transcatheter mitral valve edge-to-edge repair (MTEER) remains challenging, particularly in individuals with complex mitral valve anatomy. Conventional risk scores incorporate limited clinical variables and do not adequately account for detailed echocardiographic features, resulting in suboptimal prediction of procedural success.

**Objectives:**

To develop and evaluate machine learning (ML) and deep learning (DL) models integrating clinical and echocardiographic data to predict procedural success following MTEER.

**Methods:**

Consecutive patients undergoing MTEER at a single tertiary center between 2014 and 2022 were retrospectively analyzed. Procedural success was defined as residual mitral regurgitation ≤ mild and mean mitral valve gradient < 5 mmHg at the end of the procedure. Pre-procedural transesophageal echocardiographic (TEE) videoclips were used to generate image datasets for ML and DL model training. Machine Learning algorithms (Decision Tree, Random Forrest, Gradient Boosting, k-Nearest Neighbors, Support Vector Machines) were used to predict the primary outcome from patient demographics and baseline TEE measurements. Inception, Xception, and MobileNet architectures were evaluated using 10-fold cross-validation with strict patient-level data separation. Model performance was assessed using accuracy, sensitivity, specificity, and area under the receiver operating characteristic curve (AUC).

**Results:**

Among 467 included patients, procedural success was achieved in 90.7%. Deep learning models demonstrated superior performance compared with logistic regression and ML. The Xception architecture achieved the highest discriminatory ability (AUC 0.76), with favorable accuracy and sensitivity for predicting procedural success.

**Conclusions:**

Artificial intelligence has the potential to improve prediction of procedural outcomes following MTEER. Deep learning models that integrate echocardiographic imaging and clinical data can accurately predict procedural success and may enhance pre-procedural risk stratification, patient selection, and Heart Team decision-making.

## Introduction

Transcatheter mitral valve edge-to-edge repair (MTEER) has become an established treatment option for patients with significant mitral regurgitation (MR) who are at increased surgical risk [1–3]. Despite advances in device technology and procedural techniques, achieving optimal procedural outcomes remains highly dependent on appropriate patient selection, particularly in individuals with complex mitral valve anatomy.

Traditional surgical and transcatheter risk scores rely on a limited number of demographic and clinical variables and were not specifically developed to predict technical success of MTEER [2, 4]. Moreover, these models do not incorporate the detailed echocardiographic anatomical features that are central to procedural planning and outcome determination. As a result, their ability to guide patient selection for MTEER is limited.

Recent advances in machine learning (ML), and deep learning (DL) in particular, enable the analysis of large, high-dimensional datasets and complex, non-linear relationships between variables [5–8]. Many open-access models are uniquely suited to process imaging data and integrate it with clinical information, offering the potential to improve individualized risk prediction. In this study, we aimed to develop and evaluate AI-models based on pre-procedural echocardiographic imaging and clinical data to predict procedural success following MTEER.

## Methods

### Study population

We retrospectively analyzed all consecutive patients who underwent MTEER at St Michael’s Hospital (Toronto, Canada) between 2014 and 2022. Patients with incomplete clinical or echocardiographic data were excluded.

This study was approved by the St. Michael’s Hospital Research Ethics Board (Toronto, Ontario, Canada). The requirement for informed consent was waived by the Research Ethics Board due to the retrospective nature of the study and the use of de-identified data. The study was conducted in accordance with the ethical standards of the institutional research committee and with the Declaration of Helsinki. As this was a retrospective study using de-identified data, the requirement for informed consent was waived by the St. Michael’s Hospital Research Ethics Board. The authors received no specific funding for this work.

### Echocardiographic assessment and procedural definition

Procedures were performed using commercially available MitraClip (Abbott, Santa Clara, CA, USA) device generations available during the study period (2014–2022), in accordance with contemporary clinical practice and institutional procedural protocols. All procedures were performed under transesophageal echocardiographic (TEE) guidance using Philips Epic and IE33 ultrasound systems (Philips Healthcare, Amsterdam, The Netherlands) [9]. Standard pre-procedural TEE views were acquired, including mitral valve 2-chamber, 3-chamber, and 4-chamber views with and without color Doppler imaging. Echocardiographic guidance and post-procedural assessment was performed by three experienced interventional echocardiographers.

Immediate post-procedural echocardiographic assessment was performed by three experienced interventional echocardiographers. Procedural success was defined as residual MR ≤ mild and a mean mitral valve gradient < 5 mmHg at the conclusion of the procedure.

### AI-model development

#### Machine-learning models

Machine learning analyses were performed using five supervised learning algorithms: Decision Tree, Random Forest, Gradient Boosting, k-Nearest Neighbors (k-NN), and Support Vector Machine (SVM). A total of 34 predetermined echocardiographic measurements were selected based on their known or potential association with procedural outcomes and were used as input features for model development. Continuous variables were standardized prior to analysis. The dataset was randomly divided into training and testing cohorts, with model training performed exclusively on the training set. Hyperparameter tuning was conducted using grid-search optimization with cross-validation to identify the optimal parameters for each algorithm.

#### Deep-learning models

The DL methodology, including dataset generation and model development, has been previously described [5]. Briefly, a total of 1,374 pre-procedural TEE videoclips were extracted, corresponding to standard mitral valve views with and without color Doppler. Videoclips were converted into individual image frames, yielding a dataset of 72,952 images.

Images were labeled at the patient level as procedural success or failure based on post-procedural echocardiographic outcomes. To avoid data leakage, all images from a given patient were assigned exclusively to either the training or testing dataset.

Three convolutional neural network architectures, Inception, Xception, and MobileNet, were evaluated for binary classification. Pre-trained models were fine-tuned for the classification task. Data augmentation techniques, including resizing, rotation, flipping, and normalization, were applied to mitigate overfitting. Early stopping and batch normalization were implemented to optimize training stability.

### Model training and validation

A 10-fold cross-validation strategy was employed. In each fold, models were trained on nine subsets and tested on the remaining subset, ensuring patient-level separation between training and testing data. Model performance was evaluated using accuracy, sensitivity, specificity, and area under the receiver operating characteristic curve (AUC). As this study was designed primarily as a feasibility study to evaluate the potential of machine learning and deep learning approaches for predicting procedural success, formal statistical comparisons between individual algorithms and confidence intervals for performance metrics were not performed. Future studies using larger, multicenter datasets will be adequately powered to provide confidence intervals and statistically compare the performance of competing algorithms.

### Logistic regression model

A multivariable logistic regression model was developed as the reference statistical model for comparison with the machine learning algorithms. The model incorporated the same predefined echocardiographic variables used as input features for the machine learning models, thereby allowing direct comparison of predictive performance using identical imaging-derived information. Age, sex, and additional clinical variables were intentionally not included because the objective of the logistic regression analysis was not to develop a comprehensive clinical prediction model, but rather to evaluate the predictive value of echocardiographic features using a conventional statistical approach. Patients with incomplete echocardiographic datasets were excluded before model development; therefore, no imputation of missing values was required. Model performance was assessed using training and testing accuracy. Because the logistic regression model was developed as a benchmark classifier rather than a probabilistic prediction model, receiver operating characteristic analysis and AUC were not generated.

### Statistical analysis

Categorical variables are presented as counts and percentages, and continuous variables as mean ± standard deviation. Normality of distributions was assessed using the Kolmogorov-Smirnov test. A two-sided p-value < 0.05 was considered statistically significant. Statistical analyses were performed using Prism version 10 (GraphPad Software, San Diego, CA).

## Results

### Baseline characteristics

A total of 481 patients underwent MTEER during the study period. Fourteen patients were excluded because of incomplete data, resulting in a final cohort of 467 patients. Baseline clinical characteristics are summarized in Table 1. The mean age was 77 ± 11 years, and 44.7% of patients were female. Hypertension, atrial fibrillation, and coronary artery disease were highly prevalent. Nearly 40% of patients had a prior hospitalization for heart failure.

Baseline echocardiographic characteristics are shown in Table 2. Most patients had moderate-to-severe or severe MR, with an equal distribution of degenerative and functional etiologies. The mean left ventricular ejection fraction was 50 ± 17%.

### Procedural outcomes

**Fig. 1:**
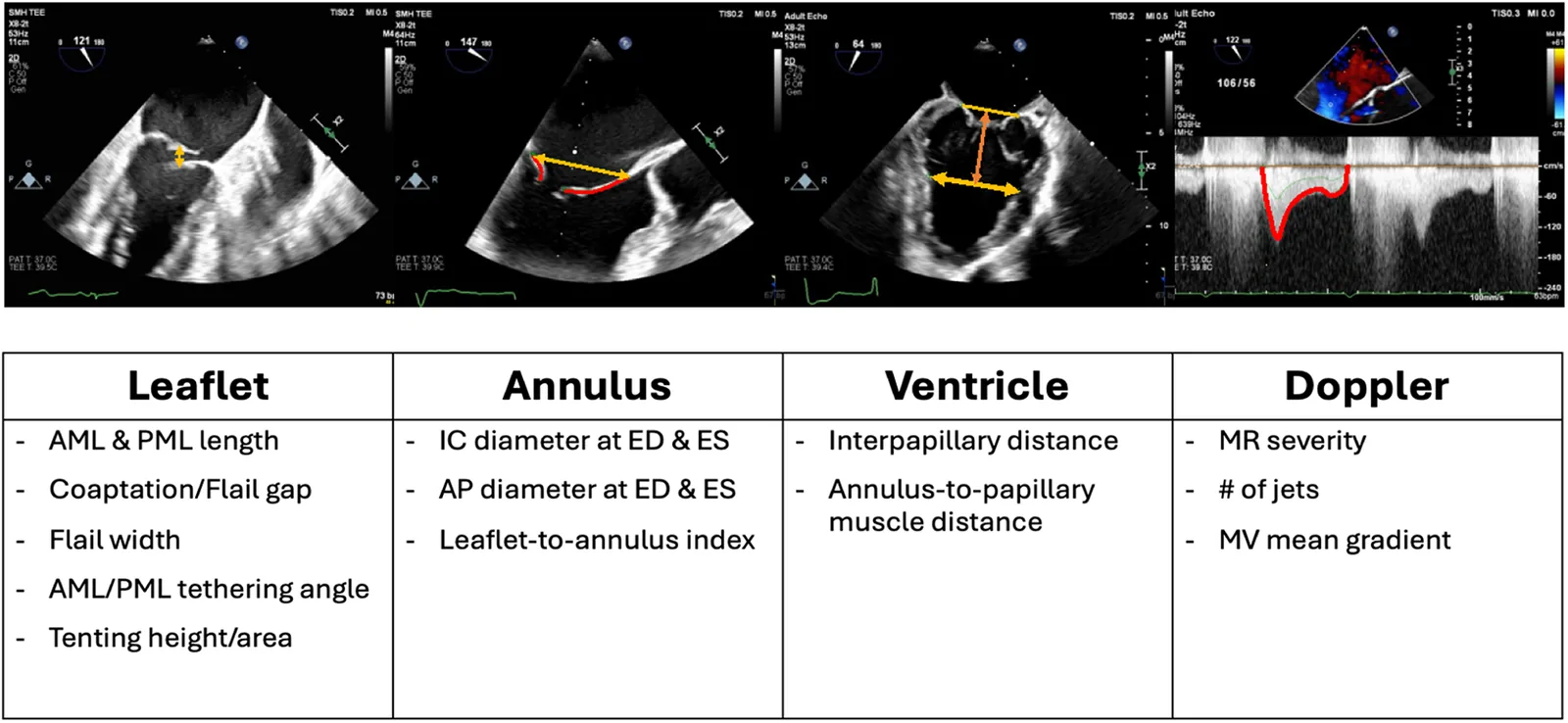
Echocardiographic parameters used for machine learning model development. Schematic representation of the echocardiographic variables used as input features for machine learning model development. Parameters were grouped into four domains: (1) leaflet morphology, including anterior and posterior mitral leaflet length, coaptation/flail gap, flail width, leaflet tethering angles, and tenting height and area; (2) annular geometry, including intercommissural and anteroposterior diameters measured in end-diastole and end-systole, as well as the leaflet-to-annulus index; (3) ventricular geometry, including interpapillary muscle distance and annulus-to-papillary muscle distance; and (4) Doppler-derived measures, including mitral regurgitation severity, number of regurgitant jets, and mean mitral valve gradient. These parameters were selected to capture the anatomical and functional determinants of procedural success following transcatheter mitral valve edge-to-edge repair (MTEER)

Procedural success was achieved in 424 patients (90.7%). A single device was implanted in 56.0% of cases, while multiple devices were required in the remaining patients. The mean post-procedural mitral valve gradient was 1.7 ± 0.9 mmHg (Fig. 1).

### Model performance

**Fig. 2:**
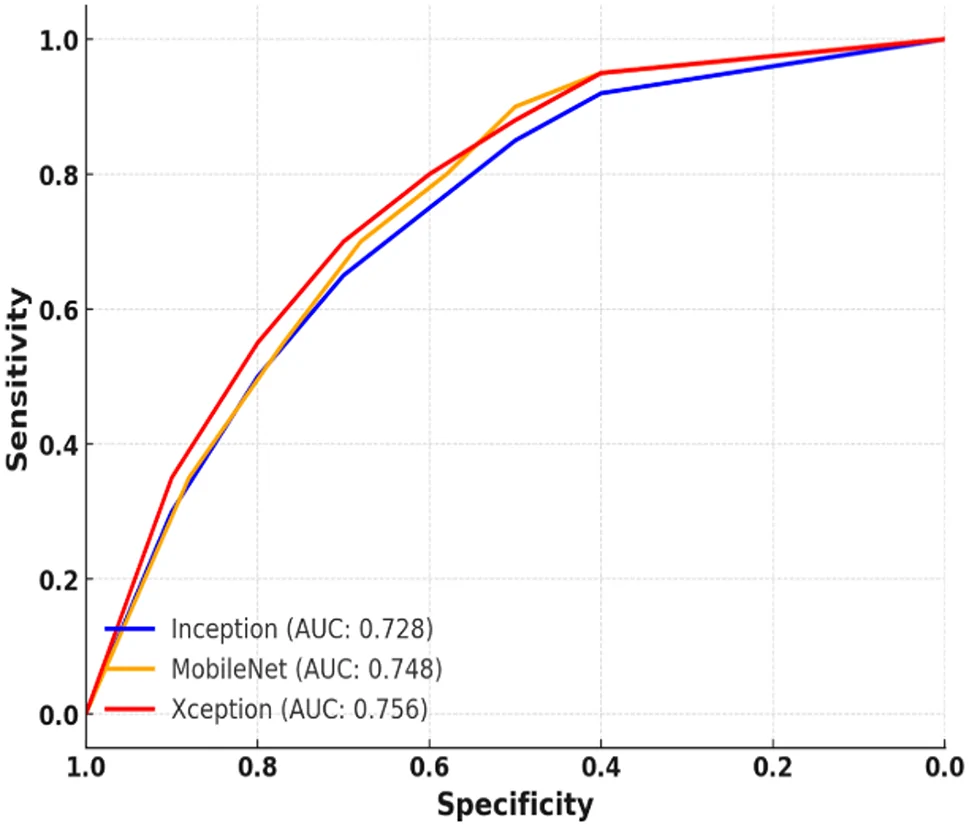
Receiver operating characteristic curves of deep learning models. Receiver operating characteristic (ROC) curves demonstrating the performance of the evaluated deep learning architectures (Inception, Xception, and MobileNet) for prediction of procedural success following transcatheter mitral valve edge-to-edge repair. The Xception model achieved the highest discriminatory performance, as reflected by the greatest area under the curve (AUC)

Machine learning model performance is summarized in Table 3. Logistic regression achieved a training accuracy of 66% and a testing accuracy of 69%. Among the machine learning algorithms, k-nearest neighbors demonstrated the highest testing accuracy (66%), followed by gradient boosting (64%), random forest (63%), and support vector machines (61%). Decision tree showed the lowest predictive performance, with a testing accuracy of 49%. Training accuracies ranged from 58% for decision tree to 66% for support vector machines. Performance metrics for logistic regression and DL models are presented in Tables 3 and 4. Deep learning models consistently outperformed logistic regression. Among the evaluated architectures, the Xception model demonstrated the highest performance, achieving an AUC of 0.76 with favorable accuracy, sensitivity, and specificity. Receiver operating characteristic curves for the deep learning models are shown in Fig. 2.

## Discussion

In this study, artificial intelligence–based approaches were feasible for predicting procedural success following MTEER. Machine learning models based on structured echocardiographic measurements demonstrated modest predictive performance, whereas DL models derived from pre-procedural TEE imaging achieved superior discrimination and outperformed conventional logistic regression. These findings suggest that image-based deep learning approaches may capture complex echocardiographic features that are not adequately represented by structured echocardiographic measurements alone.

As MTEER has become a well-established, guideline-directed therapy for patients with degenerative and functional MR who are at elevated surgical risk, accurate prediction of procedural success is increasingly important in contemporary clinical practice [3].

In real-world settings, the population treated with MTEER is considerably broader and more heterogeneous than those enrolled in pivotal randomized trials [4]. Patients often present with advanced age, multiple comorbidities, and complex mitral valve anatomy that would have excluded them from earlier studies. As a result, many centers have expanded MTEER indications to include anatomically challenging cases, such as patients with extensive leaflet or annular calcification, significant tethering, or prior surgical or transcatheter interventions [1, 10]. While this expansion has increased access to therapy, it has also introduced greater variability in procedural complexity and outcomes, underscoring the need for improved tools to predict procedural success before intervention.

Procedural success in MTEER, typically defined as residual MR less than or equal to mild and a post-procedural mean mitral valve gradient below 5 mmHg, is a critical determinant of clinical outcomes. Multiple studies have demonstrated that achieving optimal acute results is strongly associated with improved short- and mid-term outcomes, including reduced mortality and fewer heart failure hospitalizations [4, 11–12]. Data from the STS/ACC TVT Registry, which captures all commercial MTEER procedures performed in the United States, demonstrate that although most patients achieve acceptable procedural results, substantially fewer simultaneously achieve both mild-or-less residual MR and a low post-procedural transmitral gradient, a composite endpoint associated with the most favorable clinical outcomes [4]. Importantly, patients meeting both criteria experience substantially better one-year outcomes, with lower mortality (14% vs. 27%) and fewer heart failure readmissions (8% vs. 17%) than those with suboptimal procedural results. These findings highlight that procedural success is not merely a technical endpoint but a clinically meaningful goal with direct implications for patient prognosis.

Although long-term clinical outcomes, including mortality, heart failure hospitalization, and quality of life, remain the ultimate measures of treatment success, the primary objective of the present study was to predict technical procedural success using pre-procedural imaging. Immediate post-procedural residual mitral regurgitation and transmitral gradient represent technical endpoints that are directly determined by baseline mitral valve anatomy and procedural execution, making them particularly suitable targets for an imaging-based prediction model. Furthermore, optimal acute procedural results have consistently been associated with improved subsequent clinical outcomes, supporting the clinical relevance of these surrogate endpoints. Nevertheless, immediate post-procedural measurements may be influenced by transient loading conditions, general anesthesia, and intraprocedural hemodynamics, and therefore may not fully reflect longer-term valve performance or clinical status. Future studies incorporating standardized longitudinal echocardiographic follow-up and clinical outcomes will be important to determine whether artificial intelligence–based models can predict both technical and long-term therapeutic success following MTEER.

Despite the importance of procedural success, accurately predicting outcomes following MTEER remains challenging. Currently available risk scores in MR are largely derived from surgical populations and were designed primarily to estimate perioperative mortality rather than technical success of transcatheter repair [7]. These scores do not account for the nuanced echocardiographic and anatomical factors that are central to MTEER feasibility and effectiveness. Consequently, there is a substantial unmet need for risk prediction models specifically tailored to MTEER that incorporate both clinical and imaging-based parameters relevant to procedural success.

In this context, DL-based predictive models represent a promising and innovative solution [8, 13]. Deep learning has demonstrated considerable potential across multiple domains of cardiovascular medicine, particularly in its ability to analyze large, high-dimensional datasets and identify complex, non-linear relationships that are not readily captured by conventional statistical approaches. MTEER is an ideal application for DL, as procedural outcomes are influenced by a multifactorial interplay between patient characteristics, mitral valve anatomy, ventricular remodeling, and procedural factors.

One of the key advantages of deep learning is its ability to automatically extract complex image-based features directly from echocardiographic data without relying exclusively on manually predefined measurements. Unlike conventional statistical models or traditional machine learning algorithms that depend on selected variables, deep learning can identify subtle spatial relationships, leaflet morphology, tissue characteristics, and dynamic motion patterns that may not be readily apparent to human observers or adequately captured using structured echocardiographic parameters. This capability allows for a more comprehensive assessment of anatomical complexity and individualized prediction of procedural success.

In our study, DL architectures outperformed logistic regression and ML models, underscoring their superior ability to capture the complexity inherent in MTEER outcomes. The differential performance observed between ML and DL approaches likely reflects fundamental differences in how these models process echocardiographic information. The ML algorithms in this study relied exclusively on 34 predefined echocardiographic measurements selected by human operators. While these variables represent established markers of mitral valve anatomy and disease severity, they may not fully capture the complex spatial relationships, leaflet morphology, tissue characteristics, and dynamic motion patterns that influence procedural success during MTEER. In contrast, DL models were trained directly on pre-procedural TEE images and therefore had access to substantially richer information. Consequently, complex anatomical characteristics that influence procedural success, such as leaflet or annular calcification, prior mitral valve intervention, and other structural abnormalities, may have been captured indirectly through the imaging data, even when they were not explicitly included as predefined structured variables.By automatically extracting image features without requiring predefined measurements, deep learning may identify subtle anatomical patterns and interactions that are difficult to quantify using conventional echocardiographic parameters. The superior performance of deep learning compared with both ML and logistic regression suggests that a significant proportion of the predictive information relevant to MTEER outcomes resides within the imaging data itself rather than in manually derived measurements. These findings are consistent with previous studies demonstrating the ability of deep learning to uncover complex image-based phenotypes that may not be apparent to human observers or adequately represented by structured clinical variables (8).

An important methodological aspect of this study is that the analyses were performed using commercially available DL algorithms rather than institution-specific models. This approach improves transparency and reproducibility, as the algorithms are widely accessible and used in routine clinical practice. Consequently, our findings are more readily generalizable and can be independently evaluated in other clinical settings.

Another important strength of DL-based approaches is their adaptability to evolving clinical practice. MTEER technology and techniques continue to advance rapidly, with newer device generations, improved delivery systems, and refined procedural strategies. Traditional risk scores are static by nature and may become outdated as procedural paradigms change. In contrast, DL models can be retrained and updated using contemporary datasets, allowing them to maintain relevance and predictive accuracy as technologies and operator experience evolve. This adaptability is particularly valuable in the field of structural heart interventions, where innovation is continuous and rapid.

The application of DL models in MTEER also aligns closely with the broader paradigm of precision medicine. Given the substantial heterogeneity in patient anatomy, MR etiology, and comorbidity burden, a one-size-fits-all approach to patient selection is insufficient. DL-based predictive models offer the potential for individualized risk stratification, enabling Heart Teams to better identify patients who are most likely to achieve optimal procedural outcomes. Such tools could inform discussions regarding procedural feasibility, expected benefit, and alternative therapeutic strategies, thereby supporting shared decision-making and more efficient allocation of healthcare resources.

Despite these promising findings, several challenges must be addressed before deep learning–based predictive models can be widely implemented in clinical practice. High-quality, well-annotated datasets remain essential for model development and external validation, and assembling such datasets is resource intensive. Equally important is model interpretability. Although the present study focused primarily on evaluating predictive performance rather than explainability, widespread clinical adoption will require a better understanding of the imaging features driving model predictions. The commercially available deep learning framework used in this study did not readily support explainability analyses, such as saliency mapping or Gradient-weighted Class Activation Mapping (Grad-CAM), precluding identification of the specific anatomical regions contributing to model classification. Future studies should incorporate explainable artificial intelligence techniques to improve transparency, enhance clinician confidence, and facilitate integration of deep learning–based decision support into routine clinical practice. In addition, prospective external validation in large multicenter cohorts will be essential to confirm model robustness and generalizability across different patient populations, imaging protocols, and procedural practices.

Our findings suggest that DL-based predictive models have the potential to substantially enhance pre-procedural risk stratification in patients undergoing MTEER. By integrating complex echocardiographic imaging data with clinical variables, these models offer a powerful and adaptable approach to predicting procedural success, with important implications for patient selection, procedural planning, and outcomes. Continued refinement, validation, and integration of these tools into clinical workflows may represent a significant step forward in the personalized management of patients with MR.

### Study limitations

This study has several limitations. First, it was conducted at a single center, which may limit the generalizability of the findings. Although model development incorporated rigorous 10-fold cross-validation with strict patient-level separation between training and testing datasets to minimize overfitting, external validation in independent multicenter cohorts was not performed. Accordingly, the reported performance metrics should be interpreted as preliminary and may overestimate model performance when applied to external populations. Consequently, the performance of these models may differ when applied to populations with different patient characteristics, imaging protocols, operator experience, or procedural practices. Future studies should focus on external validation to confirm the robustness and generalizability of the proposed models across diverse clinical settings. Second, deep learning models are often criticized for their limited interpretability, as their decision-making processes may not be readily understandable to clinicians. While the superior performance of deep learning compared with conventional statistical and machine learning approaches suggests that these models capture complex image-based features associated with procedural success, the specific anatomical and functional characteristics driving predictions cannot be directly identified. The absence of explainability analyses, such as saliency mapping or Grad-CAM visualization, limits mechanistic insights into model behavior and represents an important area for future investigation. In addition, formal assessment of interobserver variability for echocardiographic measurements was not performed. Although all imaging was acquired and interpreted by experienced interventional echocardiographers at a high-volume structural heart center, variability in image acquisition and measurement may have influenced model inputs and performance. Furthermore, procedural outcomes were not adjudicated by an independent core laboratory, which may have introduced variability in the assessment of procedural success. In addition, the study cohort spanned an eight-year period (2014–2022), during which MTEER technology, device generations, operator experience, and procedural techniques evolved. Although this reflects real-world contemporary practice and enhances the clinical relevance of the dataset, temporal changes in procedural approaches may have influenced procedural outcomes and model performance. Future studies should evaluate model calibration and performance using contemporary cohorts and assess whether periodic model retraining is required as MTEER technologies and practice patterns continue to evolve. Finally, the primary endpoint was based on immediate post-procedural echocardiographic findings rather than longitudinal clinical outcomes. Because residual mitral regurgitation severity and transmitral gradient measured immediately after MTEER may be influenced by loading conditions and general anesthesia, these measurements may not fully reflect longer-term valve performance or clinical status. Future studies should validate these models using standardized follow-up echocardiographic and clinical endpoints.

## Conclusion

In conclusion, AI-based approaches, specifically DL models, were feasible for predicting procedural success following MTEER. Deep-learning models derived directly from pre-procedural TEE imaging achieved superior discrimination and outperformed conventional logistic regression. These findings suggest that important anatomical and functional information relevant to MTEER outcomes may be embedded within imaging data and not fully captured by conventional echocardiographic measurements. Further validation in larger multicenter cohorts is warranted to determine the clinical utility of artificial intelligence–based tools for patient selection, procedural planning, and risk stratification in contemporary MTEER practice.

**Table 1:** Baseline clinical characteristics

	Cohort (*n* = 467)
Age, years	77 ± 11
Sex female, n (%)	209 (45)
Diabetes, n (%)	118 (25)
Hypertension, n (%)	345 (74)
Smoking, n (%)	129 (28)
Dyslipidemia, n (%)	303 (65)
Chronic kidney disease, n (%)	177 (38)
CVA, n (%)	44 (9)
PVD, n (%)	25 (5)
COPD, n (%)	68 (15)
CAD, n (%)	215 (46)
Previous PCI, n (%)	113 (24)
Previous CABG, n (%)	86 (18)
Previous valvular surgery, n (%)	28 (6)
Atrial fibrillation, n (%)	299 (64)
Pacemaker, n (%)	106 (23)
Pulmonary edema, n (%)	106 (23)
Right sided-heart failure signs, n (%)	85 (18)
Previous heart failure hospitalization, n (%)	182 (39)
Weight, kg	72 ± 18
Loop diuretics, n (%)	390 (84)
Furosemide dosage, mg	65 ± 61
MRA, n (%)	151 (32)
Beta-blockers, n (%)	333 (71)
RAAS, n (%)	414 (87)
SGLT2, n (%)	25 (5)
DOAC, n (%)	252 (54)
Anti-arrhythmic, n (%)	121 (26)
NT pro-BNP, pg/mL	4279 ± 491
Creatinine, mmol/L	132 ± 81
GFR, mL/min/1.73m2	48 ± 1
Hemoglobin, g/dL	117 ± 22
Platelets, x10^9^/L	199 ± 84
STS Score. %	3.9 ± 3
Euro Score II, %	5.6 ± 4.9

**Table 2:** Baseline echocardiographic parameters

	Cohort (*n* = 467)
Degenerative MR, *n* (%)	233 (50)
Functional MR, n (%)	234 (50)
Degree of MR, n (%)	
2+ 3+ 4+	40 (9) 97 (21) 330 (71)
MVA, cm2	5.8 ± 1.5
Regurgitation volume, mL	64 ± 28
Regurgitation fraction, %	55 ± 10
Effective regurgitant orifice, cm2	0.45 ± 0.22
Vena contracta, mm	6.3 ± 4.7
PISA radius, mm	9.6 ± 2.5
Systolic to diastolic ratio	0.41 ± 0.4
LVEDD, cm	5.4 ± 1
LVESD, cm	4.0 ± 1.3
LVEDV, ml	187 ± 85
LVESV, ml	111 ± 79
LV mass index, g/m2	117 ± 38
LVEF, %	50 ± 17
Stroke volume, mL	54 ± 19
HR, bpm	74 ± 15
MV MG, mmHg	2.6 ± 1.4
LA volume index, mL/m2	67 ± 32
RVSP, mmHg	48 ± 16
TAPSE, cm	1.8 ± 0.6
TR > moderate, n (%)	91 (19)
MV intercommissural diameter diastole, cm	3.6 ± 1.9
MV intercommissural diameter systole, cm	3.3 ± 0.6
MV anteroposterior diameter diastole, cm	3.3 ± 0.2
MV anteroposterior diameter systole, cm	3.2 ± 0.5
MV annular circumference, cm2	8.7 ± 0.5
Mitral valve posterior leaflet length, cm	1.5 ± 0.6
AP/AMVL ratio	0.7 ± 0.4
AP/PMVL ratio	0.47 ± 0.4
Tenting area, cm2	0.74 ± 1.0
Tenting height, cm	0.59 ± 1.3
Coaptation/flail gap, cm	0.51 ± 0.33
Flail width, cm	0.68 ± 0.41
AMVL thickness, cm	0.23 ± 0.20
PMVL thickness, cm	0.23 ± 0.21
MV cleft, n (%)	7 (1)
MV leaflet calcification, n (%)	11 (2)
Barlow, n (%)	75 (16)
Flail, n (%)	95 (20)
Prolapse, n (%)	138 (30)
MAC, n (%)	166 (36)
MVA, cm2	4.9 ± 1.5

**Table 3:** Logistic regression and machine learning model performance

Model	Training Accuracy (%)	Testing Accuracy (%)
Logistic Regression	**66**	**69**
Decision Tree	**58**	**49**
Random Forrest	**65**	**63**
Gradient Boosting	**65**	**64**
k-Nearest Neighbors	**64**	**66**
Support Vector Machines	**66**	**61**

**Table 4:** Logistic regression and deep learning model performance

Model	Training Accuracy (%)	Testing Accuracy (%)	AUC	Sensitivity (%)	Specificity (%)
Logistic Regression	**66**	**69**			
Inception	**77.3**	**76.4**	**0.728**	**84.2**	**61.4**
Xception	**81.5**	**79.4**	**0.756**	**87.8**	**63.3**
MobileNet	**79.6**	**78.4**	**0.748**	**86.2**	**63.3**

## Data Availability

The datasets generated and/or analyzed during the current study are not publicly available because they contain protected patient health information and were collected under institutional ethics and privacy regulations that restrict public data sharing. De-identified data may be made available from the corresponding author upon reasonable request and with appropriate institutional approvals, subject to applicable privacy and ethical requirements. The machine learning and deep learning models were developed using echocardiographic measurements and imaging data obtained from patients treated at the participating institution.

## References

[1] Hausleiter J, Stocker TJ, Adamo M, et al. Mitral valve transcatheter edge-to-edge repair. EuroIntervention. 2023;18:957–76. 36688459 10.4244/EIJ-D-22-00725 PMC9869401

[2] Sorajja P, Vemulapalli S, Feldman T, et al. Outcomes with transcatheter mitral valve repair in the United States: An STS/ACC TVT Registry report. J Am Coll Cardiol. 2017;70:2315–27. 29096801 10.1016/j.jacc.2017.09.015

[3] Otto CM, Nishimura RA, Bonow RO, et al. 2020 ACC/AHA guideline for the management of patients with valvular heart disease. Circulation. 2021;143:e72–227. 33332150 10.1161/CIR.0000000000000923

[4] Mack M, Carroll JD, Thourani V, et al. Transcatheter mitral valve therapy in the United States: A report from the STS-ACC TVT Registry. J Am Coll Cardiol. 2021;78:2326–53. 34711430 10.1016/j.jacc.2021.07.058

[5] Vyas T, Chowdhury M, Xiao X, et al. Predicting mitral valve MTEER surgery outcomes using machine learning and deep learning techniques. In: Proceedings of the 9th International Conference on Mathematics and Artificial Intelligence; 2024:24–28.

[6] Johnson KW, Soto JT, Glicksberg BS, et al. Artificial intelligence in cardiology. J Am Coll Cardiol. 2018;71:2668–79. 29880128 10.1016/j.jacc.2018.03.521

[7] Topol EJ. High-performance medicine: the convergence of human and artificial intelligence. Nat Med. 2019;25:44–56. 30617339 10.1038/s41591-018-0300-7

[8] Krittanawong C, Zhang H, Wang Z, et al. Artificial intelligence in precision cardiovascular medicine. J Am Coll Cardiol. 2017;69:2657–64. 28545640 10.1016/j.jacc.2017.03.571

[9] Wunderlich NC, Siegel RJ. Peri-interventional echocardiographic assessment for the MitraClip procedure. Eur Heart J Cardiovasc Imaging. 2013;14:935–49. 24062377 10.1093/ehjci/jet060

[10] Goel K, Lindenfeld J, Makkar R, et al. Transcatheter edge-to-edge repair in patients with secondary mitral regurgitation: COAPT post-approval study. J Am Coll Cardiol. 2023;82:1281–97. 37730284 10.1016/j.jacc.2023.07.015

[11] Orban M, Rottbauer W, Williams M, et al. Transcatheter edge-to-edge repair for secondary mitral regurgitation with third-generation devices: Results from the Global EXPAND post-market study. Eur J Heart Fail. 2023;25:411–21. 36597850 10.1002/ejhf.2770

[12] Makkar RR, Chikwe J, Chakravarty T, et al. Transcatheter mitral valve repair for degenerative mitral regurgitation. JAMA. 2023;329:1778–88. 37219553 10.1001/jama.2023.7089 PMC10208157

[13] Ouyang D, He B, Ghorbani A, et al. Video-based AI for beat-to-beat assessment of cardiac function. Nature. 2020;580:252–6. 32269341 10.1038/s41586-020-2145-8 PMC8979576

